# Ice bucket challenge bears fruit for amyotrophic lateral sclerosis

**DOI:** 10.1007/s00415-016-8297-7

**Published:** 2016-10-12

**Authors:** James Hrastelj, Neil P. Robertson

**Affiliations:** Institute of Psychological Medicine and Clinical, Neurosciences, Cardiff University, Cardiff, CF14 4XW UK

## Introduction

In 2014, the ‘ice bucket challenge’ raised over $140 million worldwide for research into amyotrophic lateral sclerosis (ALS). This unprecedented boost to research funding is now beginning to deliver significant advances in our understanding of the pathogenesis of this devastating disease, and all the studies in this month’s journal club were, at least in part, funded by charitable organisations that benefited from these monies.

The first paper we discuss reports the results of the largest genetic study of ALS to date, with discovery of three new genes associated with ALS by utilising novel whole-genome sequencing technology. Importantly, the study also illuminates the genetic architecture of ALS, which in turn is likely to inform design of future genetic studies and suggests opportunities for clinical research to establish genotype–phenotype correlations. However, as genetic studies of rapidly increasing power reveal increasing numbers of risk variants and mutations, the next substantial challenge is to understand how such variants cause disease. The second and third papers discussed this month report on attempts to understand how two leading ALS risk variants cause disease and may provide exciting avenues for the development of novel therapeutic agents. The association of the *C9orf72* repeat expansion with ALS, reviewed in journal club in 2012 and 2015, is now well documented and Kramer et al. report notable progress in translating our understanding into novel therapeutic targets. Similarly, Ito et al. report that mutations in the established ALS risk gene *optineurin* may function by sensitising cells to a form of regulated cell death, which could again be targeted for therapeutic intervention.

## Genome-wide association analyses identify new risk variants and the genetic architecture of amyotrophic lateral sclerosis

Genetic sequencing technology is advancing at a considerable pace, and learning how to effectively harness the power of whole-genome sequencing will provide information of unparalleled depth and detail. Van Rheenen et al. used whole-genome sequencing data on 1246 ALS cases and 615 controls to dramatically improve the sensitivity of genetic screening to enable detection of low-frequency variants by imputation. An impressive 8.7 million SNPs were genotyped in 7763 ALS cases and 4669 controls, and by combining with genotyping data from 41 other genetic studies of ALS, a meta-analysis was performed on a total of 12,577 ALS cases and 23,475 controls. The study reports three new independent loci that associate with ALS risk with genome-wide significance: *C27orf2*, *MOBP* and *SCFD1*, which were all replicated in subsequent analysis of 2579 ALS cases and 2767 controls. Three previously associated loci were also replicated: *C9orf72*, *SARM1* and *UNC13A*.

The authors go on to report fine mapping and functional data that begin to explain how the newly discovered variants confer risk to ALS. Fine mapping of *C27orf2* in 2562 ALS cases and 1138 controls demonstrated an excess of loss-of-function mutations, suggesting pathology is caused by reduced protein product of *C27orf2*. Also, publicly available gene expression data from human brain were used to search for SNPs at the risk loci that correlate with expression of nearby genes. This analysis revealed that the variants at *SARM1* and *UNC13A* appear to, at least partly, influence function by controlling expression of the genes *POLDIP2* and *KCNN1*, respectively.

Finally, the study proceeded to analyse the genetic architecture of ALS by estimating the proportion of heritability explained by low versus high frequency variants. When compared with schizophrenia, a classical polygenic disorder dominated by common variants, the genetic risk for ALS appears to be dominated by a large number of rare variants that each confer substantial risk.

*Comment* This powerful study identifies three new loci that confer risk to ALS and provides some initial information on the possible pathogenic mechanisms. This is by far the largest genetic study in ALS to date and goes some way to resolving a fundamental question about the genetic architecture of ALS. Complementary to this work is a study published in the same edition of *Nature Genetics* by Kenna et al. who report discovery of a new gene implicated in familial ALS by exome sequencing. As a substantial proportion of the heritability of ALS is conferred by a large number of rare variants, each conferring relatively high risk to disease, future genetic studies of ALS must ensure that rare variants are captured. Such a genetic architecture implies that clinicians will have a key role in establishing genotype–phenotype correlations, which will help determine how genetic factors contribute to clinical heterogeneity.

Van Rheenen W et al. (2016) Nature Genetics 48(9):1043–1046.

## *Spt4* selectively regulates the expression of *C9orf72* sense and antisense mutant transcripts

Expansions of the hexanucleotide repeat within the *C9orf72* gene are the leading genetic cause of ALS, being responsible for 7–10 % cases of sporadic ALS and 40 % of familial cases. In a previous journal club earlier this year, we discussed a paper that explored the effect of regulation of *C9orf72* on clinical outcomes, which added to the data suggesting that the expansion caused a pathological gain-of-function. Kramer and colleagues go further in this paper by identifying a potential therapeutic avenue based on reducing the expression of *C9orf72*. Work in Huntington’s disease (HD) and on *C9orf72* suggests two major mechanisms by which such expansions can cause disease: RNA toxicity by aggregation of RNA transcribed from the repeats into intracellular foci that disrupt cellular machinery, and protein toxicity by aberrant translation of the repeat mRNA into toxic dipeptide repeat proteins (DPRs).

The authors hypothesise that *Spt4*, a transcription factor recently discovered to mediate transcription of long repeat sequences in HD, could be involved in transcription of the pathological hexanucleotide repeat expansion in *C9orf72*. The study generated three models with the pathological expansion (yeast, *C. elegans* and *Drosophila*) and found that deleting or knocking down *Spt4* reduced or eradicated RNA foci aggregation and DPR production.

The experiment was repeated in human fibroblasts isolated from ALS cases with (c9ALS) and without the *C9orf72* repeat expansion and healthy controls and detected foci of sense and antisense expansion RNA strands and DPR proteins exclusively in c9ALS fibroblasts. Using RNA interference to knock down the mammalian version of *Spt4* resulted in decreased *C9orf72* mRNA and PDR proteins, and reduced sense and antisense RNA foci. This effect was partially replicated in human cortical neurons generated from induced pluripotent stem cells.

*Comment* This study illustrates how results from genetic studies can begin to be translated into therapeutic targets. Whilst the mechanism of toxicity of the *C9orf72* expansion has not been confirmed, it is likely that treatments capable of reducing its expression will be beneficial. Targeting transcription apparatus can produce far-reaching effects on other genes, but this study also reported that the effect of reducing the expression of *Spt4* had minimal effects on the expression of other genes using RNA sequencing, suggesting that this could be a relatively specific therapeutic target.

Kramer NJ et al. (2016) Science 353(6300):708–12.

## *RIPK1* mediates axonal degeneration by promoting inflammation and necroptosis in ALS

*Optineurin* is a gene that has an established association with familial ALS, but the pathogenic mechanism is unclear. In this paper, Ito et al. report that one mechanism appears to be sensitising cells to a form of regulated cell death called necroptosis through the activity of the enzyme *RIPK1*.

The authors generated a mouse model with homozygous deletions of *optineurin*, which developed a progressive degenerative motor neuropathy and spinal cord histopathology similar to ALS. Mice that were also generated to be homozygous for inactivated *RIPK1* displayed reversed spinal cord pathology and motor dysfunction.

Several different types of mouse cells including embryonic fibroblasts, adipose cells, cultured oligodendrocytes and spinal cord samples were subject to *optineurin* deletions or knockdowns and displayed evidence of increased mediators of necroptosis including *RIPK1*. Interestingly, cultured oligodendrocytes homozygous for *optineurin* deletions underwent significantly greater cell death and this was reversed in cells also homozygous for *RIPK1* deletions or by giving an inhibitor of necroptosis (Nec-1s). In addition, microglia homozygous for *optineurin* deletions had increased levels of *RIPK1* activation and acquired a proinflammatory phenotype.

The study then investigated whether the mechanism is generalisable by investigating the *SOD1* mouse model. Markers of necroptosis were found to be raised in spinal cord samples and the mouse displayed a similar motor deficit, and both were reversed by administration of Nec-1s. Finally, analysis of spinal cord samples from human sporadic ALS cases revealed increased markers of necroptosis in microglia and oligodendrocytes.

*Comment* By investigating the functional consequences of mutations of an established risk gene, this work provides the first evidence that necroptosis may be an important pathological mechanism in ALS that could be targeted for therapeutic intervention. Interestingly, the mechanism appears to be more important in oligodendrocytes and microglia rather than neurons, which suggests that cells other than neurons may mediate at least part of the neurodegenerative process. Before pursuing potential therapeutic agents targeting necroptosis, however, further work in human cells is necessary to determine its role in neurodegeneration in familial and sporadic forms of ALS.

Ito Y et al. (2016) Science 353(6299):603–8.

